# Axillary Accessory Breast Cancer Performed Reconstruction Using a Local Flap: A Case Report

**DOI:** 10.70352/scrj.cr.26-0078

**Published:** 2026-04-01

**Authors:** Sawaka Yukishige, Norihiro Hokimoto, Keisuke Kashiwagi, Kenji Yorita, Miho Tsutsui, Hiroaki Inoue, Hiromitsu Takizawa

**Affiliations:** 1 Department of Thoracic, Endocrine Surgery and Oncology, Tokushima University Graduate School, Tokushima, Tokushima, Japan; 2 Department of Surgery, Japanese Red Cross Kochi Hospital, Kochi, Kochi, Japan; 3 Department of Plastic Surgery, Japanese Red Cross Kochi Hospital, Kochi, Kochi, Japan; 4 Department of Diagnostic Pathology, Japanese Red Cross Kochi Hospital, Kochi, Kochi, Japan; 5 Department of Pathology, Kochi Medicine School Hospital, Nankoku, Kochi, Japan

**Keywords:** accessory breast cancer, local flap, axillary reconstruction

## Abstract

**INTRODUCTION:**

Accessory breast cancer is rare, with approximately two-thirds of cases arising in the axillary region. Wide local excision is commonly performed; however, large axillary defects can lead to postoperative scar contractures and limited range of motion in the ipsilateral upper limb.

**CASE PRESENTATION:**

A 67-year-old woman presented with a palpable mass and erythema in the left axilla. Physical examination revealed a 2-cm indurated lesion with erythema in the left axilla. Ultrasonography revealed an irregular 14-mm hypoechoic mass with suspected dermal invasion. Skin punch biopsy suggested the presence of invasive carcinoma in the dermis, suggestive of invasive lobular carcinoma on immunohistochemistry. Contrast-enhanced breast MRI revealed no abnormal breast enhancement. Systemic evaluation revealed no evidence of distant metastases. The patient underwent a wide local excision of the left axillary lesion with axillary lymph node dissection, followed by local skin flap reconstruction of the axillary defect. Histopathological examination revealed an invasive lobular carcinoma of pleomorphic type (pT1cN1aM0, pStage IIA, triple-negative subtype), associated with heterotopic breast tissue and lobular carcinoma *in situ*. Postoperative adjuvant chemotherapy was administered. The patient experienced no limitations in left upper limb range of motion and survived without recurrence.

**CONCLUSIONS:**

During the surgical treatment of axillary accessory breast cancer, reconstructive procedures should be considered based on the size of the skin defect to prevent postoperative scar contracture.

## Abbreviations

ER
: estrogen receptor
FISH
: fluorescence *in situ* hybridization
GCDFP15
: gross cystic disease fluid protein 15
HE
: hematoxylin and eosin
HER 2
: human epidermal growth receptor type 2
PgR
: progesterone receptor

## INTRODUCTION

Accessory breast cancer is rare and most frequently occurs in the axillary region.^1)^ If wide axillary skin excision is required during surgery, scar contracture may limit upper limb range of motion. Here, we report a case of axillary accessory breast cancer reconstructed using a local flap.

## CASE PRESENTATION

A 67-year-old woman presented with a palpable mass and redness in the left axilla. She was referred to our dermatology department from a clinic, in which punch biopsy of the axillary skin was performed. Based on the suspicion of accessory breast cancer, the patient was referred to our department for further evaluation. The patient reported a history of swelling in the left axilla during lactation and was informed that it may represent accessory breast tissue. She had undergone mammographic screening every 2 years since 40 years of age.

Her medical history included hospitalization for acute hepatitis in her 20s. Her family history was notable for a sister with thyroid cancer, and there was no family history of breast, ovarian, or pancreatic cancers.

**Fig. 1:**
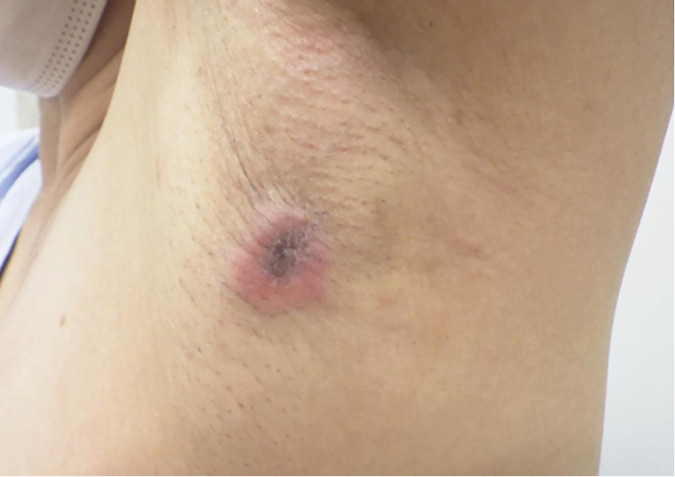
Visual and palpation examination. A firm mass accompanied by redness is palpated in the left axilla.

**Fig. 2:**
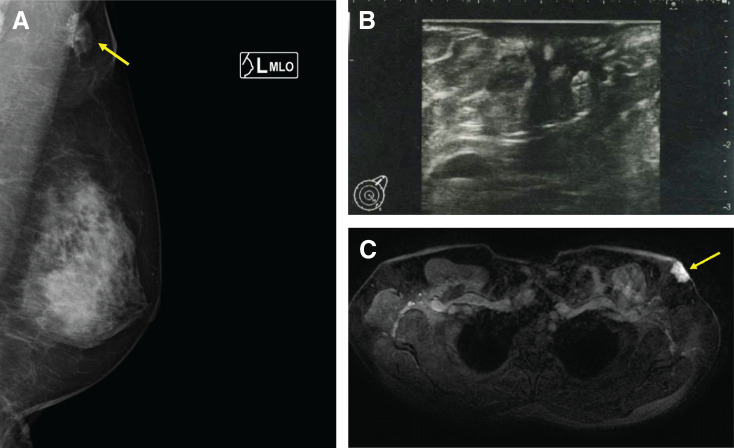
Imaging findings. (**A**) Mammography reveals a finely serrated, isodense mass (indicated by arrow) in the left axillary region. (**B**) US examination reveals an irregular hypoechoic mass measuring 14 × 12 × 11 mm with dermal invasion in the left axilla. (**C**) Breast contrast-enhanced MRI reveals a 20-mm enhancing nodule (indicated by arrow) in the left axilla.

Physical examination revealed a firm, 2.0 × 1.8 cm induration with erythema palpable in the left axilla (**Fig. 1**). No apparent accessory nipple was observed. Mammography revealed a fine, serrated, isodense mass in the left axillary region, with no abnormal findings on the right side (**Fig. 2A**). Ultrasonography revealed an irregular 14 × 12 × 11 mm hypoechoic mass in the left axilla with partially indistinct borders, posterior acoustic attenuation, internal vascularity, and suspected dermal invasion (**Fig. 2B**). No significant lymph node enlargement was observed in areas surrounding the tumor.

Histological and immunohistochemical examination of the skin punch biopsy specimen revealed dermal invasion of invasive lobular carcinoma, suggesting cutaneous invasion from the accessory breast cancer or skin metastasis from the breast cancer. Immunohistochemical staining showed cytokeratin 7 (+), cytokeratin 20 (−), GATA3 (+), GCDFP15 (+), E-cadherin (−), ER (−), PgR (−), HER2 (2+, FISH negative), and Ki-67 labeling index (12%).

Contrast-enhanced breast MRI revealed a 20-mm enhancing nodule in the left axilla (**Fig. 2C**). No abnormal enhancement areas were identified in the breasts and no obvious continuity between the intramammary glandular tissue and tumor was observed. No findings suggested the presence of accessory breast tissue in the right axilla. Whole-body evaluation using chest and abdominal contrast-enhanced CT, bone scintigraphy, and brain MRI revealed no evidence of distant metastasis. The preoperative diagnosis was left axillary accessory breast cancer (cT1cN0M0, cStage I, triple-negative subtype) and surgery was planned as the initial treatment.

**Fig. 3:**
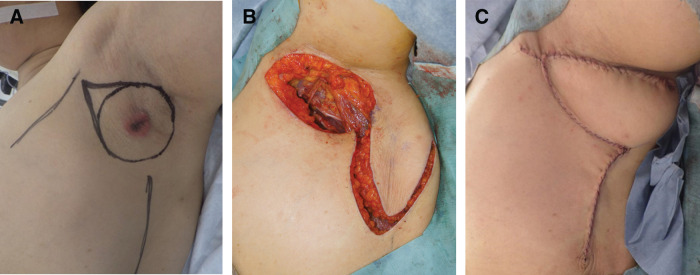
Surgical findings. (**A**) The skin excision margin is set 2 cm circumferentially from the tumor edge. (**B**, **C**) Following a left axillary wide excision and axillary lymph node dissection, the axillary defect is reconstructed using a transverse skin flap from the posterior axillary line.

The patient underwent a wide local excision of the left axillary lesion with axillary lymph node dissection, followed by local skin flap reconstruction of the axillary defect. The surgery was performed with the left upper limb slightly abducted. The skin excision margin was set 2 cm circumferentially from the tumor edge (**Fig. 3A**). To evaluate the continuity between the accessory mammary gland and normal breast tissue, a portion of the ‘C’ region of the breast was resected. ‘C’ is a Japanese Breast Cancer Society–defined designation representing the axillary tail region.

The plastic surgery team reconstructed the axillary defect using a transposition flap from the posterior axillary line (**Fig. 3B**, **3C**). The flap measured 5 × 9 cm. To avoid restricting upper arm movement when closing the donor site, it was designed along the direction of the posterior axillary line, and the flap was transposed at 90 degrees. Regarding the blood supply, perforators from the thoracodorsal vessels arising from the latissimus dorsi muscle were identified preoperatively using ultrasonography. The flap was elevated in the subfascial layer of the latissimus dorsi muscle. A suction drain was placed under the flap and was removed 5 days postoperatively. Elevation of the left upper limb was limited to the horizontal level for 1 week after surgery, after which no restrictions were imposed.

**Fig. 4:**
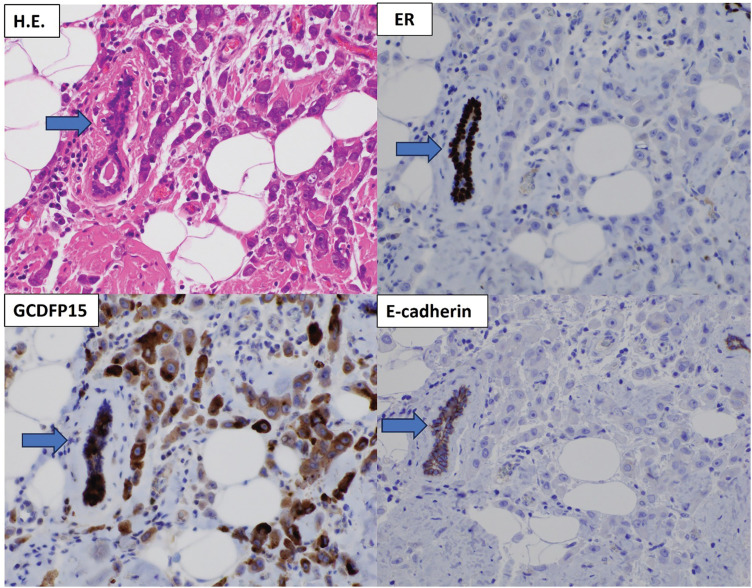
Histopathological findings of the tumor in the surgical specimens. Microscopic views of tumor sections at 400× magnification in H.E. staining revealed that atypical cells showed infiltrative proliferation in solitary or single-file arrangements. Immunohistochemical staining of the tumor cells showed ER (—), GCDFP15(+), and E-cadherin(—). Heterotopic breast duct epithelium (indicated by arrow) was positive for ER, GCDFP15, and E-cadherin. ER, estrogen receptor; GCDFP15, gross cystic disease fluid protein 15; H.E., hematoxylin and eosin

Histopathological examination of the surgical specimen revealed a 20 × 12 mm invasive lobular carcinoma of pleomorphic type (**Fig. 4**), with lobular carcinoma *in situ* (LCIS), Ly1, V0, and negative surgical margins. The immunohistochemical staining results of the tumor (**Fig. 4**) were the same as those of the skin punch biopsy. Heterotopic mammary gland tissue was identified in and around the invasive tumor (**Fig. 4**), but no histological continuity with the ‘C’ breast tissue was confirmed. The presence of LCIS and heterotopic mammary gland tissue associated with the invasive carcinomas suggests primary accessory breast cancer. The postoperative diagnosis was left axillary accessory breast cancer, pT1cN1a (3/12)M0, pStage IIA.

**Fig. 5:**
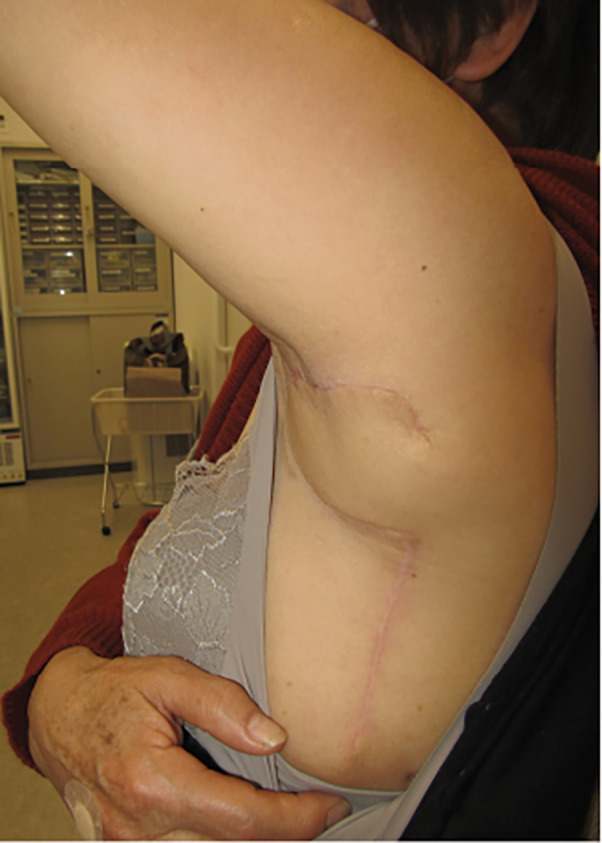
Postoperative course. This photograph is taken 1 year after surgery. There is no blood flow impairment in the skin flap, and elevation of the left upper limb is possible without any issues.

As adjuvant therapy, the patient received chemotherapy comprising four cycles of epirubicin and cyclophosphamide (EC) followed by four cycles of nab-paclitaxel (nab-PTX). Radiotherapy was not administered. At the latest follow-up (46 months postoperatively), the patient remained disease-free without recurrence. No obvious postoperative complications were observed. The range of motion of the left shoulder joint was unchanged from the preoperative status and was comparable to that of the contralateral side (**Fig. 5**).

## DISCUSSION

Accessory breast cancer is rare, accounting for approximately 0.2%–0.6% of all breast cancers, with approximately two-thirds arising in the axillary region.^2,3)^ It infiltrates the surrounding tissues at an early stage and has a relatively high incidence of axillary lymph node metastasis.^4)^ Cutaneous manifestations appear early, and previous reports have described skin erythema, fixation, and ulceration in approximately 60% of cases.^5)^

The diagnostic criteria for accessory breast cancer include the following: (1) exclusion of metastatic carcinoma from other organs, (2) presence of non-cancerous mammary gland tissue surrounding the lesion without continuity with the orthotopic breast, and (3) exclusion of tumors derived from similar structures, such as sebaceous or sweat glands.^6)^ In the present case, preoperative imaging confirmed the absence of primary lesions in other organs and within the breasts. During surgery, a portion of the ‘C’ region of the breast was resected to assess continuity, and pathological examination revealed no connection with the orthotopic breast tissue. Therefore, accessory breast cancer was diagnosed. The presence of LCIS and heterotopic breast tissue associated with the invasive carcinoma supported the diagnosis of a primary lesion arising from the ectopic mammary tissue.

In most cases of accessory breast cancer, a wide local excision of the lesion with axillary lymph node dissection is performed.^7)^ Because the lymphatic drainage of accessory breast tissue has not been fully elucidated and axillary lymph node metastasis is frequently observed,^8)^ this case was diagnosed as cN0; however, axillary lymph node dissection was performed instead of sentinel lymph node biopsy.

If the resulting axillary skin defect is extensive, direct closure may lead to scar contracture, restricting shoulder movements on the affected side.

In the present case, the patient had axillary accessory breast cancer with skin involvement, and a relatively large skin defect was expected to result in an adequate surgical margin. Primary closure within the axilla alone would cause significant tension, leading to impaired arm elevation, and skin grafting alone carries a high risk of postoperative scar contracture.^5)^ Therefore, reconstruction of the axillary defect was planned to prevent contracture. We provided adequate skin replacement, created axillary mobility, and preserved arm elevation by transferring a skin flap from the posterior axillary line, in which there was sufficient skin laxity. This type of flap is known as a transposition flap. Postoperatively, the patient showed no limitation in the upper limb range of motion, indicating that the reconstructive procedure was beneficial.

In previously reported cases of axillary accessory breast cancer in Japan, 12 cases underwent reconstruction. The reconstruction methods included latissimus dorsi myocutaneous flaps in 5 cases,^3–5,9,10)^ parascapular flaps in 4,^11–14)^ local flaps in 2,^15,16)^ and a free flap in 1.^17)^ All the cases in which local flaps were used, including this case, were classified as cT1. Because local flaps are insufficient for larger defects, reconstruction with a latissimus dorsi flap or other options should be considered for T2 or larger tumors.

Although the surgical margins were negative in this case, invasive lobular carcinoma is known to exhibit a diffuse growth pattern; therefore, careful assessment of the margins was required. If the margins had been positive, adjuvant radiation therapy would have needed to be considered. Local flap reconstruction is generally regarded as being able to tolerate postoperative radiotherapy, which is also considered an advantage of this reconstructive approach.

For curative resection, an adequate surgical margin must be secured; however, this can result in a large skin defect and carries a high risk of restricting upper-limb mobility. Therefore, axillary defect reconstruction is crucial in maintaining oncological curability and patient QOL.

## CONCLUSIONS

Reconstruction using a local flap is useful for axillary accessory breast cancer. To prevent scar contracture, reconstructive procedures should be considered based on the size of the axillary defect.

## DECLARATIONS

### Availability of data and materials

The datasets supporting the conclusions of this article are included within the article.
